# Effectiveness and Safety of Avatrombopag in Liver Cancer Patients with Severe Thrombocytopenia: Real-World Data and Challenges

**DOI:** 10.1155/2022/9138195

**Published:** 2022-11-09

**Authors:** Ao Huang, Jia-Feng Chen, Jian-Zhang Wu, Zheng Gao, Ying-Hong Shi, Xiu-Tao Fu, Xin Zhang, Wei-Ren Liu, Qiang Gao, Hui-Chuan Sun, Guo-Ming Shi, Jia Fan, Zhen-Bin Ding, Jian Zhou

**Affiliations:** ^1^Department of Liver Surgery and Transplantation, Zhongshan Hospital, Fudan University, Shanghai 200032, China; ^2^Liver Cancer Institute, Zhongshan Hospital, Fudan University, Key Laboratory of Carcinogenesis and Cancer Invasion (Fudan University), Ministry of Education, Shanghai 200032, China; ^3^Department of Gastrointestinal & Pancreatic Surgery, Zhejiang Provincial People's Hospital, Key Laboratory of Gastroenterology of Zhejiang Province, Hangzhou, Zhejiang 310014, China; ^4^Institute of Biomedical Sciences, Fudan University, Shanghai 200032, China; ^5^State Key Laboratory of Genetic Engineering, Fudan University, Shanghai 200032, China; ^6^Shanghai Xuhui Central Hospital, Zhongshan-Xuhui Hospital, Fudan University, Shanghai 200031, China

## Abstract

**Background:**

Avatrombopag has been approved in patients who have severe thrombocytopenia (<50 × 10^9^/L) and chronic liver disease (CLD) while receiving invasive procedures. The real-world application and effectiveness of avatrombopag in the subgroup patients with liver cancer remain unknown.

**Methods:**

Liver cancer patients (including primary liver cancer and colorectal cancer liver metastasis) who had severe thrombocytopenia and received avatrombopag were retrospectively enrolled. Avatrombopag dose, peak and absolute platelet count increase, combination treatment with other thrombopoietic agents, responder (peak count ≥50 × 10^9^/L with absolute increase ≥20 × 10^9^/L) rate, and anticancer treatment effect were analyzed. Thrombosis and bleeding events were assessed.

**Results:**

In total, 93 patients were enrolled, with 72 and 21 in the CLD and non-CLD groups, respectively. Patients with CLD had hepatitis B or C, larger spleen volume, and a higher cirrhosis degree. Baseline platelet counts were similar between two groups (median, 37.0 × 10^9^/L *vs.* 39.0 × 10^9^/L; *P*=0.594), while patients without CLD had higher peak platelet (median, 134.0 × 10^9^/L *vs*. 74.0 × 10^9^/L; *P*=0.015) and absolute increase (median, 101.0 × 10^9^/L *vs*. 41.0 × 10^9^/L; *P*=0.020) after avatrombopag treatment. The responder rate was higher in patients without CLD (100% *vs*. 76.4%; *P*=0.010). Combined avatrombopag with other thrombopoietic agents significantly increased platelet count; repeated use of avatrombopag produced similar effects with that of initial treatment. Concerning anticancer treatment effect, patients who responded to avatrombopag had a higher disease control rate. No thrombosis or hemorrhagic events were observed, even in patients with portal vein tumor thrombosis.

**Conclusion:**

Avatrombopag was safe and effective and ensured successful implementation of anticancer treatment in liver cancer patients with severe thrombocytopenia, accompanied with or without CLD.

## 1. Introduction

Thrombocytopenia is common in patients with chronic liver disease (CLD) and worsens with the degree of liver cirrhosis [1, 2]. This situation is more complicated in liver cancer, which is the sixth most frequent cancer types and the fourth leading cause of cancer-related mortality globally because invasive therapeutic procedures [3], including hepatectomy, radiofrequency ablation, and biopsy, carry additional hemorrhage risk. Besides, for patients who receive systemic therapy, thrombocytopenia is not a rare event due to myelotoxicity or immunotherapy-related myelosuppression [4–6], resulting in chemotherapy dose reduction, prolonged interval between treatment cycles, or shifting to less-intensive regimens with the risk of a decreasing response rate and overall survival. Under such circumstances, thrombopoietic drugs, such as corticosteroids and recombinant human thrombopoietin, or platelet transfusion are often prescribed [7]. However, due to liver cirrhosis and hypersplenism, such drugs are not able to timely, effectively, and steadily increase platelet count, and platelet transfusion is not always available due to donor shortage. Thus, it is necessary to investigate novel agents to increase platelet count in a rapid and lasting manner to prevent hemorrhagic events during anticancer treatment [8].

Based on the outcomes of the ADAPT-1 and ADAPT-2 trials, avatrombopag, a second-generation thrombopoietin receptor agonist (TPO-RAs), which activates the c-Mpl TPO receptor and stimulates proliferation and differentiation of megakaryocytes [9], has been approved by the US FDA for treatment of patients with thrombocytopenia and CLD who need to undergo an invasive procedure [10, 11]. Avatrombopag has a favorable pharmacological profile with similar exposure in patients of different countries and races [9, 12], and in April 2020, avatrombopag was also approved by the National Medical Products Administration in China, being effective and safe in the management of thrombocytopenia in Chinese patients with CLD [12]. To date, there is little evidence of avatrombopag in liver cancer patients with thrombocytopenia and it is unknown whether the actual treatment effect of avatrombopag is as effective as that in the ADAPT-1 and ADAPT-2 trials. Thus, it is necessary to evaluate the real-world application of avatrombopag in these patients, especially in China, where most liver cancer patients (mainly hepatocellular carcinoma, HCC) have CLD resulting from hepatitis B virus (HBV) infection [13]. In addition, off-label use of avatrombopag in liver cancer patients without CLD is not rare in clinical practice, which also deserves attention.

In the current study, we evaluated the real-world data of avatrombopag in liver cancer patients with thrombocytopenia in order to describe the characteristics of these patients, evaluate their clinical outcomes including platelet count increase and thrombotic or bleeding events, and characterize treatment patterns including duration of therapy and attainment of a treatment-free period. We also evaluated the anticancer treatment response indirectly contributed by the thrombopoietic effect of avatrombopag.

## 2. Methods

### 2.1. Patient Selection

**Figure 1:**
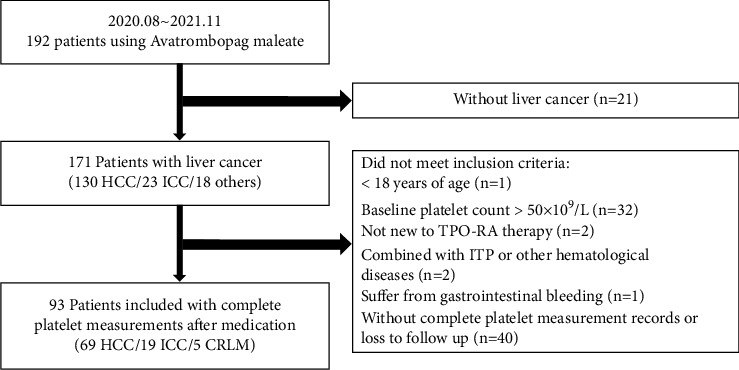
Flowchart of the patient enrollment process. HCC: hepatocellular carcinoma; ICC: intrahepatic cholangiocarcinoma; CRLM: colorectal cancer liver metastasis; TPO-RA: thrombopoietin receptor agonist; ITP: immune thrombocytopenia.

The flow chart in Figure 1 describes the patient selection process. The major inclusion criteria were patients with liver cancer, aged ≥ 18 years, and platelet count <50 × 10^9^/L at the baseline. All patients had given informed consent to participate in the study. From August 2020 to November 2021, a total of 192 patients who received avatrombopag and were treated in the Liver Cancer Institute, Zhongshan Hospital, Fudan University, were retrospectively reviewed. CLD is caused by the etiologies of alcohol abuse, metabolic disorders, and autoimmune and chronic viral hepatitis. CLD is marked by gradual destruction of liver parenchyma and deterioration of liver function for more than six months [14]. Twenty-one patients without liver cancer were excluded, and 78 patients who did not meet the inclusion criteria or had no detailed follow-up information were subsequently ruled out, leaving 93 patients enrolled in the study. There were 69 patients with HCC, 19 with intrahepatic cholangiocarcinoma (ICC), and five with colorectal cancer liver metastasis (CRLM). Baseline patient characteristics were retrieved from the medical records. The spleen volume was calculated using the data measured from enhanced magnetic resonance imaging (MRI): spleen volume index (SI) = *W* × *T* × *L* (W, spleen maximal width; *T*, thickness; *L*, length) [15, 16]. Fibroscan values were measured by ultrasound elastography in 55 patients (44 CLD and 11 non-CLD patients) to assess liver stiffness. The albumin-bilirubin (ALBI) score was calculated as log_10_ total bilirubin (*μ*mol/L) × 0.66 + albumin (g/L) × −0.085 [17]. This study was approved by the Research Ethics Committee, Zhongshan Hospital Fudan University.

### 2.2. Thrombopoietic Treatment and Platelet Measurement

Avatrombopag (Doptelet; Fosun Pharma) was prescribed according to the indication: patients were given 5 days of avatrombopag, 60 mg daily if the baseline platelet count was <40 × 10^9^/L or 40 mg daily if it was 40×10^9^–50×10^9^/L. Upon completion of avatrombopag administration, the platelet count was monitored every 2–4 days from day 6 to the day before anticancer treatment and a couple of times after anticancer treatment. The platelet counts were recorded from laboratory results in the medical records. Peak platelet counts were the highest counts during the avatrombopag treatment period while absolute platelet count increase was calculated as peak platelet count minus baseline platelet count, as previously reported [18]. Platelet transfusion, hemorrhagic events requiring rescue procedures, and venous thrombosis during or after avatrombopag treatment were recorded. The responder was defined as peak platelet count ≥50 × 10^9^/L with absolute platelet count increase ≥20 × 10^9^/L from the baseline.

### 2.3. Anticancer Treatment and Treatment Response Evaluation

The anticancer treatment was made according to the tumor pathology and stage of liver cancer. For HCC, the China Liver Cancer Staging System was used to guide treatment [19], which included hepatectomy, ablation, transcatheter arterial chemoembolization (TACE), targeted therapy, and immunotherapy. For ICC, the TNM staging system was used [20]. For CRLM, hepatectomy and chemotherapy combined with targeted therapy were the main choices. Liver biopsy was performed if the treatment was needed to be decided on the basis of tumor pathology and genotyping. All patients were monitored by routine blood tests, including tumor markers every 4–8 weeks up to March 2022. Enhanced MRI or computed tomography (CT) of the abdomen was performed every 3 months to assess radiological response. Evaluation of anticancer treatment response was made using the mRECIST criteria.

### 2.4. Statistical Analysis

All statistical analyses were performed using SPSS software (IBM Corp.), and *P* < 0.05 was considered to be statistically significant. Continuous variables were summarized by median and range, while categorical variables were summarized by number and frequencies. To test the differences between the groups, chi-square or Fisher's exact test for categorical variables and the Mann–Whitney *U* test for continuous variables were used. Parameters with *P* < 0.1 in univariate analysis were enrolled in the multivariate linear regression model to identify the potential parameters associated with the avatrombopag treatment outcome.

## 3. Results

### 3.1. Demographics and Baseline Laboratory Parameters

The final analysis included 93 liver cancer patients who had severe thrombocytopenia and received avatrombopag. Patients were classified into the CLD group (*n* = 72) or non-CLD group (*n* = 21). The baseline demographic characteristics and pretreatment laboratory results of the two groups were summarized in Table 1. No difference in gender was observed between the two groups, and patients with CLD tended to be younger than those without CLD (median, 53.5 *vs*. 62.0 years; *P*=0.026). Patients with CLD caused by hepatitis B or C were more frequently associated with liver cirrhosis, which was reflected by larger splenic volume (*P*=0.007) and higher fibroscan value (*P*=0.116). Other clinical and laboratory factors were similar between the two groups.

### 3.2. Efficacy and Safety of Avatrombopag in Liver Cancer Patients

**Figure 2:**
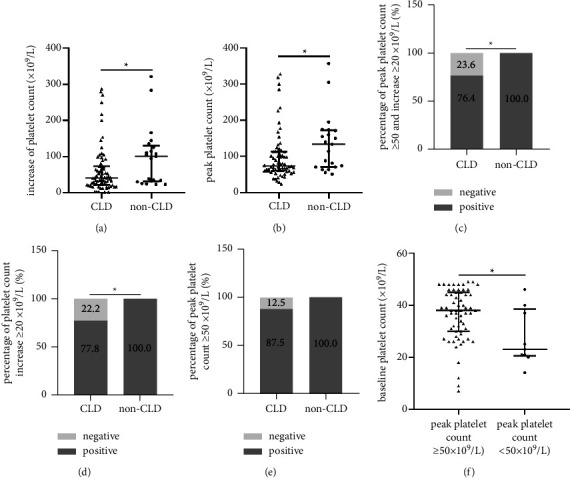
Thrombopoietic effect of avatrombopag in liver cancer patients with and without CLD. (a) Scatter plot of peak platelet count in the CLD and non-CLD groups after avatrombopag treatment; (b) scatter plot of absolute platelet increase in the CLD and non-CLD groups after avatrombopag treatment; (c) percentages of responders after avatrombopag treatment in the CLD and non-CLD groups; (d) percentages of patients whose absolute platelet increased ≥20×109/L after avatrombopag treatment in the CLD and non-CLD groups; (e) percentages of patients who had peak platelet count ≥50×109/L after avatrombopag treatment in the CLD and non-CLD groups; (f) scatter plot of baseline platelet count of patients with peak platelet count ≥50 × 109/L or <50 × 109/L after avatrombopag treatment in CLD group. CLD: chronic liver disease.

Avatrombopag significantly increased the absolute platelet count in all patients with a median increase of 41.0 × 10^9^/L and produced 81.7% responders (Table 2). Subgroup analysis showed that the baseline platelet count was similar between the CLD and non-CLD groups (median, 37.0 × 10^9^/L *vs*. 39.0 × 10^9^/L; *P*=0.594) (Table 2). Better thrombopoietic effect of avatrombopag was observed in patients without CLD since the absolute platelet count increase (Figure 2(a)), peak platelet count (Figure 2(b)), percentage of responders (Figure 2(c)), and percentages of patients with absolute platelet count increase ≥20 × 10^9^/L (Figure 2(d)) were all higher in the non-CLD group than in the CLD group. Although no significant difference was found in the percentage of patients with peak platelet count ≥50 × 10^9^/L (a criterion that does not require platelet transfusion) between the two groups, all patients in the non-CLD group had reached the target count, but nine patients had failed in the CLD group (Figure 2(e), Table 2). Moreover, the rate of responders to avatrombopag was also higher in patients without CLD (100% *vs.* 76.4%; *P*=0.010). Scrutiny of these nine patients found that they had lower baseline platelet count when compared with the other patients in the CLD group (median, 23.0 × 10^9^/L *vs*. 38.0 × 10^9^/L; *P*=0.017; Figure 2(f)). Most patients (90.3%) maintained the target platelet count ≥50 × 10^9^/L before anticancer treatment, indicating a steady and lasting thrombopoietic effect of avatrombopag.

Throughout the treatment period, no deep venous or portal venous thrombosis occurred, even in the 12 patients who had portal vein tumor thrombosis (PVTT) and in another eight patients who had peak platelet >200 × 10^9^/L. Avatrombopag was well tolerated, and no symptomatic adverse effects were observed during its administration. No hemorrhagic complications that required rescue therapy were observed, and only two events of platelet transfusion occurred during or after the procedures in patients who received surgery or other types of invasive procedures. Four patients ceased systemic treatment or immunotherapy/targeted therapy due to refractory thrombocytopenia, even though they received avatrombopag.

### 3.3. Factors Influencing the Platelet Increase the Effect of Avatrombopag

To identify patients who could benefit from treatment with avatrombopag, we analyzed which clinicopathological factors were associated with better absolute platelet count increase (Table 3). Although patients with CLD were younger, age did not influence the outcome of avatrombopag treatment. Patients who had viral hepatitis (median, 41.0 × 10^9^/L *vs*. 101.0 × 10^9^/L; *P*=0.020) and larger splenic volume (median, 34.5 × 10^9^/L *vs*. 54.0 × 10^9^/L; *P*=0.018) were less likely to have higher platelet increase, while patients who received combination therapy with other thrombopoietic agents had higher absolute platelet count increase than those who did not (median, 69.0 × 10^9^/L *vs*. 35.0 × 10^9^/L; *P*=0.012). In addition, there was no significant difference in absolute platelet count increase between patients with different baseline platelet count (median, 39.0 × 10^9^/L *vs*. 42.0 × 10^9^/L; *P*=0.275).

**Figure 3:**
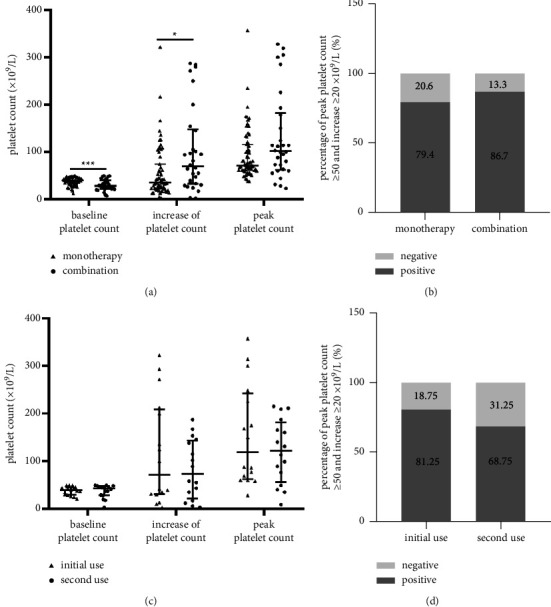
Thrombopoietic effect of combined avatrombopag with other thrombopoietic drugs and repeated use in liver cancer patients. (a) Thrombopoietic effect of avatrombopag in patients with and without combination treatment; (b) percentages of responders in patients with and without combination treatment; (c) thrombopoietic effect of patients with initial and second use of avatrombopag; (d) percentages of responder in patients with initial and second use of avatrombopag.

We then used multiple linear regression analysis to verify these results (Supplementary Table 1). Combination with other thrombopoietic agents including thrombopoietin or recombinant human interleukin-11 (rhiL-11) significantly enhanced the outcome of avatrombopag treatment (*P*=0.006), while splenectasis hindered the increase in platelet count, although the difference was only marginally significant (*P*=0.055). We thus thoroughly investigated the effect of combination treatment of avatrombopag with other thrombopoietic drugs to increase platelet count. Thirty patients received thrombopoietin or rhiL-11 as combination treatment, and the percentages of combination treatment were similar between the CLD and non-CLD groups (33.3% *vs*. 28.6%; *P*=0.794) (Table 1). Compared with patients who received avatrombopag monotherapy, these patients had lower baseline platelet count (median, 28.0 × 10^9^/L *vs*. 39.0 × 10^9^/L; *P*=0.001), larger splenic volume (median, 1216.8 *vs*. 910.0 cm^3^; *P*=0.200), and an inferior child-Pugh score (median, 6.0 *vs*. 5.0; *P*=0.004) (Supplemental Table 2). Although combination treatment promoted absolute platelet increase, as a result of lower baseline platelet count, the peak platelet count did not differ between the two groups (Figure 3(a)). The percentage of responders was also similar between the two groups (Figure 3(b)).

### 3.4. Treatment Response of Repeated Use of Avatrombopag

Avatrombopag was prescribed on days 1–5 and invasive procedures were scheduled 5–8 days after the final dosage. However, not all patients could successfully maintain the increased platelet count after the scheduled treatment and repeated (≥2 times before the invasive procedure or during anticancer treatment) use of avatrombopag is not rare in clinical practice. Indeed, sixteen patients had repeated administration of avatrombopag, and seven of them had received avatrombopag ≥3 times. Baseline platelet counts were similar between initial and second use (median, 38.5 × 10^9^/L *vs*. 39.0 × 10^9^/L; *P*=0.724) (Figure 3(c)). A comparable thrombopoietic effect was observed between second use and initial treatment with avatrombopag, in terms of absolute platelet count increase (median, 71.0 × 10^9^/L *vs*. 73.5 × 10^9^/L; *P*=0.696) and peak platelet count (median, 119.0 × 10^9^/L *vs*. 122.0 × 10^9^/L; *P*=0.468). Although the percentage of responders tended to be higher in patients at initial treatment, the difference was not significant (*P*=0.685) (Figure 3(d)).

### 3.5. Platelet Count Enhanced by Avatrombobag Optimized Anticancer Response

**Figure 4:**
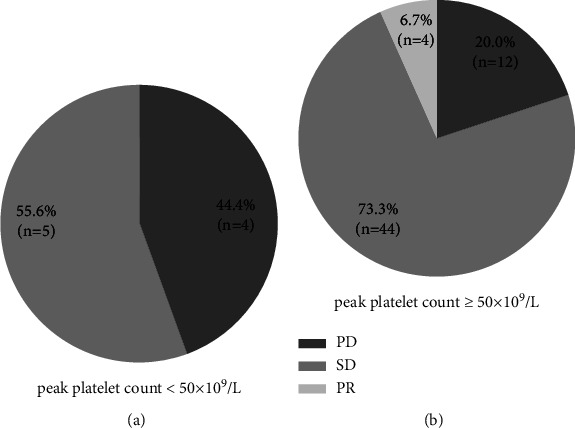
Comparison of anticancer response between patients with different peak platelet counts after avatrombopag treatment. PD: progressive disease; SD: stable disease; PR: partial response.

We finally analyzed whether avatrombopag contributed to the anticancer treatment response in patients with HCC. The 69 HCC patients were grouped on the basis of peak platelet count ≥50 × 10^9^/L after avatrombopag treatment, and a tendency towards higher percentages of stable disease and partial response (80.0% *vs*. 55.6%, *P*=0.227) was noted in patients who had responded to avatrombopag (Figure 4). In the 9 patients whose platelet count did not reach 50 × 10^9^/L after avatrombopag, four had ceased anticancer treatment due to severe and refractory thrombocytopenia and subsequently experienced disease progression.

**Figure 5:**
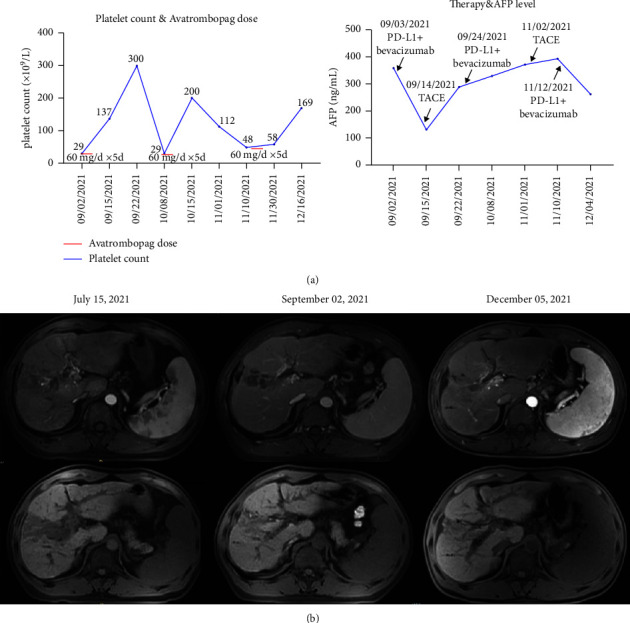
One typical case of the patient who successfully finished the scheduled anticancer treatment plan with the thrombopoietic effect of avatrombopag. (a) Platelet count, avatrombopag dose, the AFP level, and anticancer treatment over time; (b) changes in enhanced MRI of the patient with HCC.

We presented a typical case of a patient who successfully completed the scheduled anticancer treatment plan with the thrombopoietic effect of avatrombopag. A 44-year-old male patient with HCC and PVTT on a background of HBV-related liver cirrhosis received TACE, portal vein stent, and iodine 125 radioactive particle therapy as initial treatment. One month later, he received combination treatment of antiangiogenic therapy (bevacizumab; Avastin) plus anti-PD-L1 antibody (atezolizumab; Tecentriq). Upon the second cycle treatment, the low platelet count was a contraindication and avatrombopag was prescribed. After avatrombopag (60 mg daily) for 5 days, the patient experienced a dramatic increase in platelet count, which peaked at 300 × 10^9^/L, and he successfully underwent the second TACE combined with bevacizumab + atezolizumab. In the following treatment, low platelet count occurred for another two times and each time, avatrombopag helped increase platelet count to a level that ensured implementation of anticancer treatment without hemorrhage risk (Figure 5(a)). The patient survived till now and maintained stable disease status (Figure 5(b)).

## 4. Discussion

Thrombocytopenia is not rare in patients with CLD, occurring in 64%–84% of patients with cirrhosis or fibrosis. It could be a potentially treatment-limiting adverse event of particular interest in liver cancer patients, delaying necessary invasive procedures of diagnostic or therapeutic intent, necessitating targeted agent dose reduction or treatment discontinuation, resulting in suboptimal treatment outcomes [21, 22]. For liver cancer patients without CLD, myelosuppression, which is related to anticancer treatment such as chemotherapy, radiotherapy, immunotherapy, and targeted therapy, could also result in thrombocytopenia, and sometimes, it is necessary to cease therapy to ensure a safe platelet count to avoid bleeding risk.

Thrombopoietic agents and prophylactic platelet transfusions are suggested for these patients. However, traditional thrombopoietic agents such as recombinant human thrombopoietin are not effective enough to increase platelet count in a short period of time, failing to ensure timely implementation of invasive procedures. Besides, platelet transfusion is complicated by the risk of transfusion-related reactions, unknown infection, allergy, and most of all, a shortage of donors [23]. Moreover, the invasive therapeutic procedures are often repeatedly performed in these liver cancer patients. Thus, the efficacy of platelet transfusion is likely to be limited because repeated transfusion may induce refractoriness to subsequent transfusion [24]. Alternative medications to promote effective as well as rapid platelet increase remain an unmet need in these patients [25].

In recent years, avatrombopag was approved for CLD patients with thrombocytopenia undergoing invasive procedures. In this study, we analyzed the real-word data of liver cancer patients who received avatrombopag and restricted the enrolled patients to those with platelet count <50 × 10^9^/L at the baseline, the same as that in the ADAPT-1 and ADAPT-2 trials. We found that the treatment effect of avatrombopag was satisfactory since 87.5% of patients with CLD achieved the target platelet count ≥50 × 10^9^/L and did not require platelet transfusion or any rescue procedures. This was even better than the outcomes in the ADAPT-1 and ADAPT-2 trials. Avatrombopag treatment in our study produced 76.4% responders in liver cancer patients with CLD, which was superior to that reported in the phase II clinical randomized study from Japan [26]. The increased platelet count sustained until the day of the scheduled invasive procedure or during anticancer treatment, and no adverse event of thrombosis was observed, even in patients who had high peak platelet count and PVTT. Such a result might be attributed to the fact that avatrombopag increases only platelet count and does not activate platelets [27]. Consistent with our results, a recent study reported successful treatment with avatrombopag in two patients bearing urological neoplasms who developed anti-PD1 antibody-induced amegakaryocytic thrombocytopenia and were refractory or intolerant to any other thrombopoietic agents including glucocorticoids, cyclosporine, intravenous immunoglobulin, rh-thrombopoietin, and eltrombopag [28]. Thus, the present study reproduced the results of clinical trials in the real-world setting, and avatrombopag was considered effective and safe for liver cancer patients with severe thrombocytopenia.

Although treatment of liver cancer patients without CLD is not the indication, avatrombopag has been explored for new clinical indications [9] and prescribed in response to the demand for rapid platelet increase in clinical practice. Indeed, it displayed better thrombopoietic effects in these patients than in patients with CLD, and this result coincided with the fact that patients with viral hepatitis and larger splenic volume had lower platelet count increase. In fact, avatrombopag is effective for treatment of chemotherapy-induced severe and refractory thrombocytopenia in patients with solid tumors but without CLD [29]. Such an increase in platelet count reduces the need for platelet transfusions and enables chemotherapy dose intensity to be maintained [30]. However, this does not mean avatrombopag should be routinely considered for those patients since anticancer treatment-related myelosuppression could be treated without much difficulty in practice and the cost-effectiveness of avatrombopag should be weighed [31, 32], especially considering the serious adverse effects, including deep vein or portal vein thrombosis, during or after avatrombopag treatment. In line with this notion, a randomized, double-blind, placebo-controlled, phase 3 trial of avatrombopag in patients with nonhematological cancer and chemotherapy-induced thrombocytopenia reported similar proportions of patients who reached the primary endpoint in the avatrombopag and placebo groups [33]. Thus, caution should still be exercised in patients without CLD despite the impressive thrombopoietic effect in these patients.

Different from clinical trials, we observed quite a few patients who received combination treatment of avatrombopag with other thrombopoietic agents, and indeed, the combination treatment showed a better effect in increasing platelet count. In fact, patients who received combination treatment had lower baseline platelet count and more serious CLD. This may explain the choice of combination treatment: initial treatment with conventional agents was not ideal in this group of patients, and avatrombopag was subsequently introduced to increase platelet count. Although TPO-RA mimics the biological effect of TPO *in vitro* and *in vivo*, the two kinds of agents could act simultaneously on the TPO receptor, which might be responsible for the additive thrombopoietic effect [34]. This is the first time that combined avatrombopag with other thrombopoietic agents has cooperative effect *in vivo* in the treatment of thrombocytopenia, which was consistent with the *in vitro* results that AKR-501 (avatrombopag) in combination with TPO had an additive effect on megakaryocytopoiesis [34].

Another important finding is that patients who repeatedly received avatrombopag had similar platelet count increase with that of the initial treatment. In this study, 16 patients received avatrombopag ≥2 times, and the repeated use of avatrombopag was also effective and safe, showing similar efficacy to the initial treatment without additional thrombotic risk. For the first time, avatrombopag has been reported to be repeatedly used in clinical practice. For liver cancer patients, invasive procedures, such as locoregional therapies, might be performed several times and each time, patients should be evaluated for platelet count and bleeding risk. The reproducible, rapid, and durable thrombopoietic effects of avatrombopag make it ideal for these patients.

While this study had a number of informative findings and certainly added to the current evidence base, it indeed had some limitations. Even though we had made strict eligibility criteria in the research design, the innate defects of real-world design, namely, the results were mostly descriptive, the absence of a control group, and the risk of systematic errors should be seriously considered. Besides, controlling for all possible confounding variables in routine clinical practice is challenging. Thirdly, the sample size was relatively small since the patients who had used avatrombopag were heterogenous, and a part of them had been excluded according to our criteria. Therefore, the outcomes of this study should be interpreted with caution, and inference of causal relationships should be avoided. Despite these limitations, the real-world data presented here enriched our experience with this population of liver cancer patients who were receiving avatrombopag, and the effectiveness and safety of avatrombopag in real-world clinical practice, both on- and off-label use, aligned with results of previous randomized controlled trials.

In conclusion, our real-world data suggested that avatrombopag was effective for liver cancer patients with severe thrombocytopenia and CLD. The off-label use of avatrombopag in liver cancer patients without CLD displayed an impressive thrombopoietic effect. Avatrombopag was a safe and effective alternative to platelet transfusion, simplifying the clinical management of thrombocytopenia in liver cancer patients with or without CLD, minimizing hemorrhage risk, and safeguarding anticancer treatment.

## Figures and Tables

**Table 1:** The characteristics of enrolled liver cancer patients grouped by the presence or absence of chronic liver disease.

Characteristics	Total (*n* = 93)	CLD (*n* = 72)	Non-CLD (*n* = 21)	*P* value^#^
Age (years)	56.0 (29.0∼87.0)	53.5 (29.0∼87.0)	62.0 (42.0∼85.0)	0.026^†^
Sex (male/female)	68/25	55/17	13/8	0.188^*∗*^
Etiology (HBV/HCV/nonviral)	67/5/21	67/5/0	0/0/21	<0.001^*∗*^
Hemoglobin (g/L)	121.0 (59.0∼170.0)	121.0 (59.0∼170.0)	120.0 (62.0∼139.0)	0.358^†^
Albumin (g/L)	39.0 (24.0∼52.0)	38.0 (24.0∼52.0)	42.0 (25.0∼48.0)	0.410^†^
TB (*μ*mol/L)	18.5 (5.0∼177.2)	19.2 (5.0∼177.2)	17.7 (5.2∼96.0)	0.748^†^
ALT (U/L)	27.0 (7.0∼332.0)	27.0 (7.0∼332.0)	31.0 (14.0∼69.0)	0.377^†^
AST (U/L)	38.0 (14.0∼244.0)	37.5 (14.0∼244.0)	38.0 (21.0∼82.0)	0.432^†^
ALBI score	−2.5 (−3.6∼−0.98)	−2.5 (−3.6∼−0.98)	−2.7 (−3.4∼−1.25)	0.607^†^
Child-Pugh score	6.0 (5.0–11.0)	5.5 (5.0∼11.0)	6.0 (5.0–11.0)	0.741^†^
Fibroscan value (kPa)	15.1 (6.0∼25.0)	15.7 (6.0∼25.0)	13.5 (6.0∼18.0)	0.116^†^
Spleen volume index (cm^3^)	1116.6 (351.7∼3566.6)	1244.1 (351.7∼3566.6)	751.7 (392.9∼1649.4)	0.007^†^
Combination with TPO or rhIL-11 (P/N)	30/63	24/48	6/15	0.681^*∗*^

Values are median (range); ^#^*P* value of the comparison between the CLD and non-CLD groups; †Mann–Whitney *U* test; ^*∗*^Pearson *χ*.^2^ tests or Fisher's exact test, as appropriate; abbreviations: CLD: chronic liver disease; HBV: hepatitis B virus; HCV; hepatitis C virus; TB: total bilirubin; ALT: alanine transaminase; AST: aspartate transaminase; ALBI score: albumin-bilirubin score; TPO: thrombopoietin; rhIL-11: recombinant human interleukin-11; P: positive; N: negative.

**Table 2:** Thrombopoietic effect of avatrombopag in liver cancer patients grouped by the status of chronic liver disease.

	Total (*n* = 93)	CLD (*n* = 72)	Non-CLD (*n* = 21)	*P* value^#^
Baseline platelet count (×10^9^/L)	37.0 (7.0∼49.0)	37.0 (7.0∼49.0)	39.0 (19.0∼49.0)	0.594†
Peak platelet count (×10^9^/L)	80.0 (23.0∼357.0)	74.0 (23.0∼328.0)	134.0 (51.0∼357.0)	0.015†
Median increase of platelet count (×10^9^/L)	41.0 (2.0∼322.0)	41.0 (2.0∼287.0)	101.0 (23.0∼322.0)	0.020†
Increase of platelet count ≥20 × 10^9^/L (P/N)	77/16	56/16	21/0	0.019^*∗*^
Peak platelet count ≥50 × 10^9^/L (P/N)	84/9	63/9	21/0	0.201^*∗*^
Peak platelet count ≥50 × 10^9^/L and Increase ≥20 × 10^9^/L (P/N)	76/17 (81.7%)	55/17 (76.4%)	21/0 (100%)	0.010^*∗*^

Values are median (range); ^#^*P* value of the comparison between the CLD and non-CLD groups; †Mann–Whitney *U* test; ^*∗*^Pearson *χ*.^2^ tests or Fisher's exact test, as appropriate; abbreviations: CLD: chronic liver disease; P: positive; N: negative.

**Table 3:** Clinicopathological factors associated with platelet count increase effect of avatrombopag.

Factors	Number (%)	Platelet count increase	*P* value
Age (years)			
≤55	46 (49.5)	42.0 (2.0∼287.0)	0.738
>55	47 (50.5)	41.0 (2.0∼322.0)	

Gender			
Male	68 (73.1)	40.0 (2.0∼287.0)	0.102
Female	25 (26.9)	45.0 (17.0∼322.0)	

Etiology			
HBV or HCV	72 (77.4)	41.0 (2.0∼287.0)	0.020
Nonviral	21 (22.6)	101.0 (23.0∼322.0)	

Child-Pugh Grade			
A	70 (75.3)	41.0 (2.0∼322.0)	0.682
B or C	23 (24.7)	42.0 (3.0∼284.0)	

Combination with TPO or rhIL-11			
Positive	30 (32.2)	69.0 (2.0∼287.0)	0.012
Negative	63 (67.7)	35.0 (2.0∼322.0)	

Spleen volume index (cm^3^)			
≤1000	41 (44.1)	54.0 (2.0∼322.0)	0.018
>1000	52 (55.9)	34.5 (2.0∼287.0)	

Baseline platelet count (×10^9^/L)			
<40	37 (39.8)	42.0 (2.0∼322.0)	0.275
≥40	56 (60.2)	39.0 (2.0∼287.0)	

ALBI score			
≤−2.6	44 (47.3)	35.5 (2.0∼322.0)	0.131
>−2.6	49 (52.7)	45.0 (2.0∼287.0)	

Values are median (range) and compared by the Mann-Whitney*U* test; abbreviations: HBV: hepatitis B virus; HCV: hepatitis C virus; TPO: thrombopoietin; rhIL-11: recombinant human interleukin-11; P: positive; N: negative; ALBI score: albumin-bilirubin score.

## Data Availability

Data could be acquired from the corresponding author upon reseasonable request.

## Supplementary Materials

Supplementary Materials Supplementary Table 1: multiple linear regression identified factors relevant with the platelet count increase effect of avatrombopag. Supplemental Table 2: the characteristics of patients with or without combination treatment.
