# Supplementary material for: Effectiveness and Safety of Avatrombopag in Liver Cancer Patients with Severe Thrombocytopenia: Real-World Data and Challenges
Source: J Oncol. 2022 Nov 9;2022:9138195. doi: 10.1155/2022/9138195 (PMC9668468; doi:10.1155/2022/9138195)
Supplement: Supplementary Materials — Supplementary Table 1: multiple linear regression identified factors relevant with the platelet count increase effect of avatrombopag. Supplemental Table 2: the characteristics of patients with or without combination treatment. [file 9138195.f1.docx]

**Effectiveness and safety of avatrombopag in liver cancer patients with severe thrombocytopenia: Real-world data and challenges**

Ao Huang^1, 2, #^, Jia-Feng Chen^1, 2, #^, Jian-Zhang Wu^3^, Zheng Gao^1, 2^, Ying-Hong Shi^1, 2^, Xiu-Tao Fu^1, 2^, Xin Zhang^1, 2^, Wei-Ren Liu^1, 2^, Qiang Gao^1, 2^, Hui-Chuan Sun^1, 2^, Guo-Ming Shi^1, 2^, Jia Fan^1, 2, 4, 5^, Zhen-Bin Ding^1, 2, 6, *^, Jian Zhou^1, 2, 4, 5, 6, *^

1. Department of Liver Surgery and Transplantation, Zhongshan Hospital, Fudan University, Shanghai, 200032, China.
2. Liver Cancer Institute, Zhongshan Hospital, Fudan University; Key Laboratory of Carcinogenesis and Cancer Invasion (Fudan University), Ministry of Education, Shanghai, 200032, China.
3. Department of Gastrointestinal & Pancreatic Surgery, Zhejiang Provincial People’s Hospital, Key Laboratory of Gastroenterology of Zhejiang Province, Hangzhou, Zhejiang, 310014, China.
4. Institute of Biomedical Sciences, Fudan University, Shanghai, 200032, China.
5. State Key Laboratory of Genetic Engineering, Fudan University, Shanghai, 200032, China.
6. Shanghai Xuhui Central Hospital, Zhongshan-Xuhui Hospital, Fudan University, Shanghai, 200031, China.

^#^, These authors contributed equally to this work.

^*^, Corresponding Author: Jian Zhou, Liver Cancer Institute, Zhongshan Hospital, Fudan University, 136 Yi Xue Yuan Road, Shanghai, 200032, China, E-mail: zhou.jian@zs-hospital.sh.cn； or Zhen-Bin Ding, Liver Cancer Institute, Zhongshan Hospital, Fudan University, 136 Yi Xue Yuan Road, Shanghai, 200032, China. E-mail: ding.zhenbin@zs-hospital.sh.cn

**Supplementary Table 1.** Multiple linear regression identified factors relevant with platelet count increase effect of avatrombopag

| **Factor** | **B（95%CI）** | ***P* value** |
| --- | --- | --- |
| Combination with TPO or rhIL-11（P/N） | 42.462 (12.697~72.226) | 0.006 |
| Etiology (HBV or HCV/Non-viral) | -28.747 (-63.144~5.650) | 0.100 |
| Spleen volume index | -0.022 (-0.044~＜0.001) | 0.055 |

Abbreviations: HBV, hepatitis B virus; HCV, hepatitis C virus; TPO, thrombopoietin; rhIL-11, recombinant human interleukin-11; P, positive; N, negative.

**Supplemental Table 2.** The characteristics of patients with or without combination treatment

| **Characteristic** | **Combination with TPO or rhIL-11 (n=30)** | **Monotherapy of avatrombopag (n=63)** | ***P* value** |
| --- | --- | --- | --- |
| Age (years) | 53.5 (29.0~87.0) | 54.0 (29.0~85.0) | 0.931† |
| Sex (Male/Female) | 22/8 | 46/17 | 0.974* |
| Etiology  (HBV/HCV/Non-viral) | 23/1/6 | 44/15/4 | 0.793* |
| Hemoglobin (g/L) | 103.0 (59.0~141.0) | 127.0 (66.0~170.0) | ＜0.001† |
| Albumin (g/L) | 36.0 (24.0~45.0) | 42.0 (30.0~52.0) | ＜0.001† |
| TB (μmol/L) | 21.8 (5.0~177.2) | 16.7 (5.2~96.0) | 0.044† |
| ALT (U/L) | 29.0 (7.0~332.0) | 26.0 (9.0~288.0) | 0.660† |
| AST (U/L) | 38.0 (14.0~244.0) | 38.0 (17.0~171.0) | 0.721† |
| ALBI score | -2.0 (-3.0~-0.98) | -2.7 (-3.6~-1.7) | ＜0.001† |
| Child-Pugh score | 6.0 (5.0~11.0) | 5.0 (5.0~10.0) | 0.004† |
| Fibroscan value (kPa) | 15.6 (10.0~21.0) | 15.0 (6.0~25.0) | 0.476† |
| Spleen volume index (cm^3^) | 1216.8  (363.5~3566.6) | 910.0  (351.7~2944.0) | 0.200† |
| Baseline platelet count (×10^9^/L) | 28.0 (7.0~49.0) | 39.0 (12.0~49.0) | 0.001† |

Values are median (range);

†Mann-Whitney *U* test; *Pearson χ^2^ tests or Fisher’s exact test, as appropriate;

Abbreviations: TPO, thrombopoietin; rhIL-11, recombinant human interleukin-11; HBV, hepatitis B virus; HCV, hepatitis C virus; TB, total bilirubin; ALT, alanine transaminase; AST, aspartate transaminase; ALBI score, albumin-bilirubin score.
